# Using atomic force microscopy for physical virology: touching and manipulating single virus particles

**DOI:** 10.1007/s12551-026-01407-0

**Published:** 2026-01-30

**Authors:** Alejandro Díez-Martínez, Klara Strobl, A. Cámara-Ballesteros, R. Delgado-Buscalioni, Pedro José de Pablo

**Affiliations:** 1 Departamento de Física de la Materia Condensada and IFIMAC, Madrid, 28049 Spain; 2 Departamento de Física Teórica de la Materia Condensada and IFIMACl, Madrid, 28049 Spain

**Keywords:** Atomic force microscopy, Force curve, Nanoindentation, Beam deflection, Tip, Cantilever, Stylus, Topography, Aqueous solution, Mechanical fatigue, DNA condensation, Assembly, Disassembly

## Abstract

Atomic force microscopy (AFM) employs a nanometer-scale tip mounted on a microcantilever to scan surfaces where virus particles have been captured. Beyond generating high-resolution images of individual virions in liquid, AFM offers unique capabilities: manipulation of single particles, investigation of their biomechanical properties, and real-time observation of assembly and disassembly processes, including genome release. This chapter begins by outlining fundamental aspects of virus adsorption and imaging, highlighting, among other factors, the influence of tip-convolution artifacts. These principles are applied to reveal the adsorption behavior of the TGEV coronavirus on surfaces. Subsequent sections detail approaches for probing TMV’s mechanical properties through single-indentation experiments and mechanical fatigue protocols. In this section, the mechanical fatigue approach is also discussed when used on 2D arrays of viral coat proteins. The review also discusses how these mechanical techniques can trigger genome release in minute virus of mice (MVM), a process that can alternatively be induced by temperature, as happens in bacteriophage T7. Finally, the chapter illustrates how AFM can serve as a nanomanipulation tool to move individual viruses across surfaces and estimate their adhesion strength.

## Introduction

Viruses exemplify how natural evolution can create highly sophisticated systems using minimal resources (Bamford and Zuckerman 2021). They function as nanoscale machines whose properties are not only crucial for understanding viral biology but also hold promise for applications in materials science (Rother et al. 2016). In this context, the mechanical behavior of biomolecular complexes is fundamental to their function, and viruses are no exception (Mateu 2013). The metastability of viral structures allows the precise modulation of their physicochemical properties to accomplish each stage of the infection cycle. While enveloped viruses store and transport their genome in a lipidic container, non-enveloped viruses are primarily composed of a capsid (a protein shell formed by repeating subunits—capsomers) that enclose their viral genome (Bamford and Zuckerman 2021). Far from being static, both enveloped and non-enveloped viruses are dynamic assemblies capable of shuttling and delivering their genome between hosts through an automated process. These naturally evolved features have inspired the use of viral capsids as protein containers for artificial cargoes such as drugs, polymers, enzymes, and minerals, with applications spanning biomedicine and materials science (Wang and Douglas 2021).

Whether natural or engineered, virus cages must protect their cargo against harsh physicochemical conditions, including molecular collisions in crowded environments (Zhou et al. 2008), thermal and chemical stress (Agirre et al. 2011), and osmotic shocks (Cordova et al. 2003). Therefore, it is essential to employ techniques that provide insights into the stability of these structures under varying conditions and track their structural changes. Structural biology methods such as electron microscopy (EM) and X-ray crystallography reveal high-resolution architectures of protein cages (Baker et al. 1999), but they rely on averaging data from millions of particles (X-ray) or thousands of structures (cryo-EM). Consequently, these approaches offer limited information on particle-to-particle variability and require non-physiological conditions (e.g., vacuum), preventing real-time characterization of virus shell dynamics in liquid environments.

The emergence of single-molecule techniques has practically demonstrated that the mechanical properties of biological assemblies are critical to their function (Egan et al. 2015). Exploring these properties complements traditional structural biology by uncovering the interplay between structure, function, and mechanics. Atomic Force Microscopy (AFM) not only enables imaging of individual protein-based particles in liquid but also provides physicochemical data for each particle (Pablo 2024). Furthermore, AFM’s nano-dissection capabilities allow the local manipulation of protein shells, offering insights into their assembly and disassembly processes (Pablo and San Martin 2022). This review starts by illustrating how the imaging abilities of AFM provide experimental data for feeding theoretical simulations that unveil the adhesion mechanisms of coronavirus on surface (García-Arribas et al. 2024). Afterwards, we explain how AFM can be employed to measure the biomechanics of tobacco mosaic virus (TMV) and its implications on virus stability and disassembly (Díez-Martínez et al. 2025). The subsequent topic deals with the AFM study of ssdNA release from individual minute virus of mice (MVM) (Strobl et al. 2023) and the thermal ejection of dsDNA from bacteriophage T7. We also illustrate how the nanotribology of individual viruses helps to understand the adhesion strength of viruses on surfaces (Ault et al. 2025). Finally, pulling experiments with AFM are also discussed.

## Broad adaptability of coronavirus adhesion

**Fig. 1:**
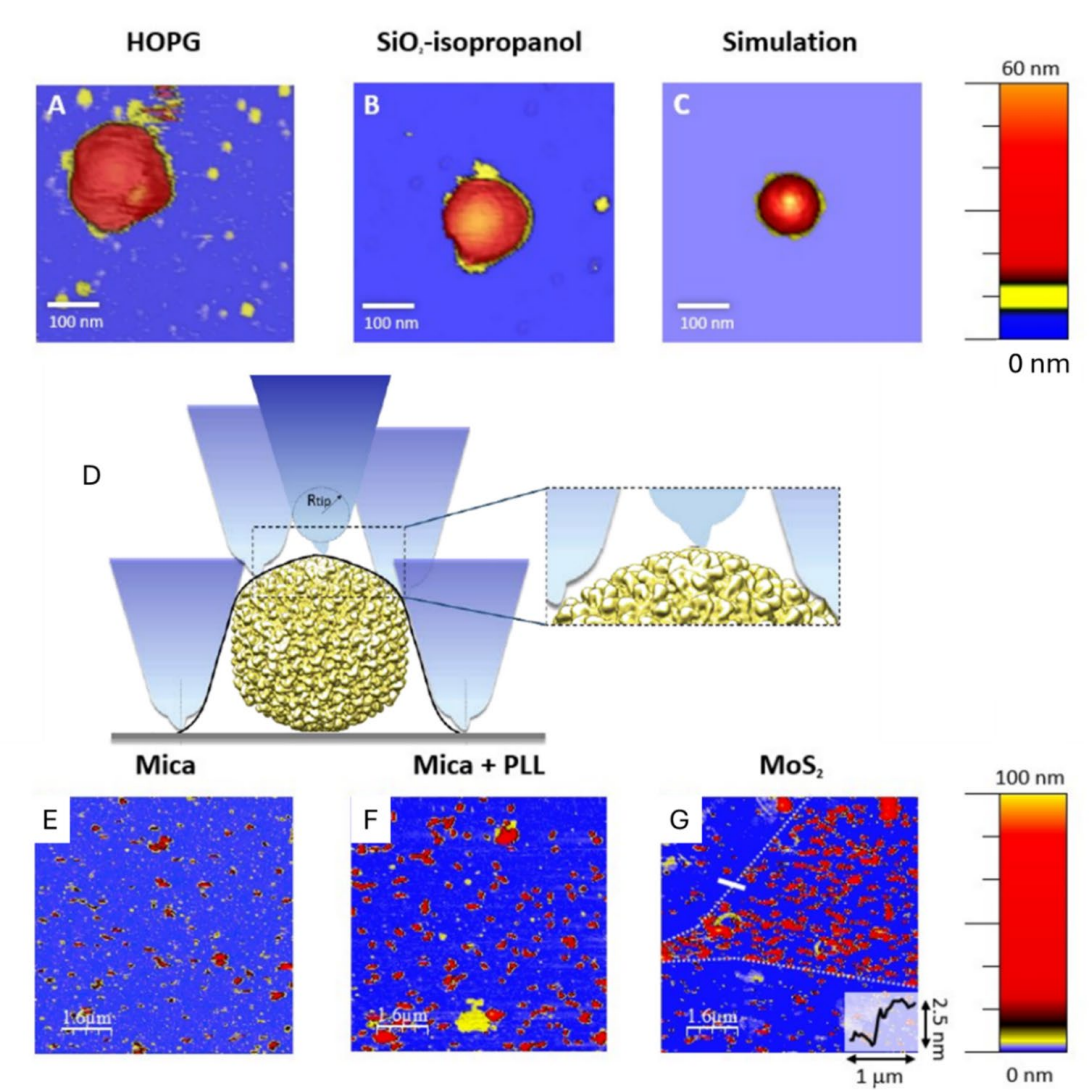
Examples of AFM topographical images of TGEV on various substrates. Panels **A** (HOPG) and **B** (SiO_2_‐isopropanol) illustrate single virus particles highlighting their nearly spherical shape due to lipid envelopes. The image size is ≈500 × 500 nm^2^. Panels **A** and **B** correspond to real virus with radii 53 and 50 nm, respectively. Panel **C** shows the virtual AFM image of a coarse‐grained virus model (with average radius R v ≈ 43 nm). In panel **C**, the structural details in yellow are the adsorbed viral spikes. **D** Cartoon showing the dilation effect of the AFM tip. Adapted from (Llauró-Portell 2016). Panels **E** (mica), **F** (mica + PLL), and **G** (MoS_2_) exhibit images with a 1/30 viral dilution on each surface. Panel **F** includes a dotted white line to highlight MoS_2_ ‐ SiO_2_ boundaries, and the inlet caption shows a cross section of the solid white line, displaying the ≈ 2.5-nm thickness of the MoS_2_ flake. Adapted from (García-Arribas et al. 2024)

**Fig. 2:**
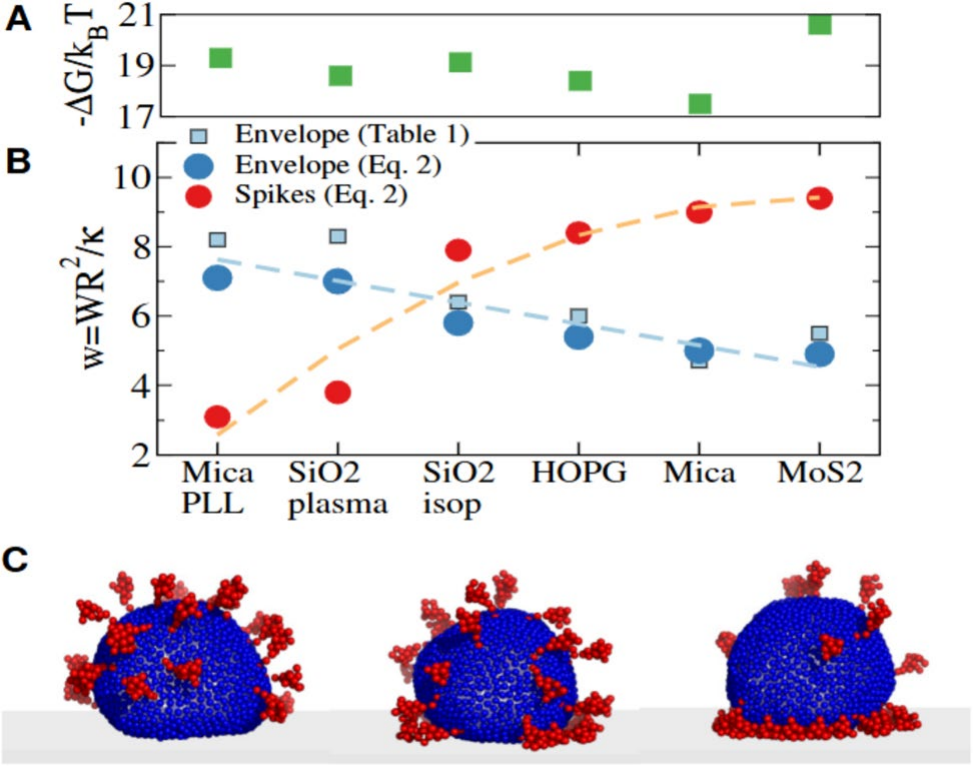
Illustration of the different conformations of the coronavirus according to the spike and membrane adhesion at each surface. **A** Experimentally measured adhesion free energy − ΔG/k_B_ T. **B** Circles indicate the values of the energy for capturing the spikes and the lipid shell. Squares correspond to energy of the capsid envelope derived from the analysis of AFM profiles. Dashed lines, drawn to guide the eye, highlight the opposite trends for the lipid membrane and spikes energies. **C** Coarse grain simulations for a case with large membrane adhesion (left), large spike adhesion (right), and similar adhesion (middle). Adapted from (García-Arribas et al. 2024)

Inanimate surfaces can act as fomites for disease transmission (Castaño et al. 2021), while new materials are being developed to enhance virus capture and inactivation (Zhang et al. 2022). Understanding virus–surface interactions is crucial for biological and health contexts (Joonaki et al. 2020). The adhesion free energy (ΔG), which reflects viral affinity for a surface, can be derived from the dissociation constant. This depends on interfacial free energy per area (W) and viral mechanical properties such as surface tension (Σ) and bending rigidity (κ), both key factors in the adhesion of enveloped viruses (Zeng et al. 2017). The transmissible gastroenteritis virus (TGEV) is a swine coronavirus which shares with SARS-CoV-2 most of the structural features (Kiss et al. 2021; Kubo et al. 2022; Cantero et al. 2022). SARS-CoV-2 and TGEV present a similar compression rigidity or stiffness as measured with AFM (k ≃ 0.01 N/m). Due to the strong similarities, TGEV is one of the most frequently used surrogates for SARS-CoV (Bárcena et al. 2009; Roldan-Hernandez and Boehm 2023) and can be used to study the adhesion of coronavirus (CoV) structures on surfaces. Thus, AFM can be employed to study the capture of TGEV coronavirus on different reference surfaces by measuring the topographies of adsorbed viruses (Lecot et al. 2021), contact angles with the surface and virus surface coverage (trapping). These surfaces include mica, mica pretreated with poly-l-lysine (“mica + PLL”), highly oriented pyrolytic graphite (HOPG), isopropanol-cleaned silicon oxide (SiO_2_-isopropanol), and plasma-cleaned silicon oxide (SiO_2_-plasma), and a bicomponent surface composed of SiO2 and Molybdenum disulfide, MoS_2_. Using APTES was not contemplated because it did not work due to the lipid nature of enveloped viruses. In spite of its pleomorphic nature, TGEV can be ascribed a radius of R = (41 ± 2) nm (Kiss et al. 2021; Domingo and Faraudo 2021). TGEV is decorated with ≈ 40 spikes of 15–20 nm in length (Domingo and Faraudo 2021; Ke et al. 2020) which are remarkably mobile and flexible (Turoňová et al. 2020; Luo et al. 2023). AFM offers relevant information about the surface virus capture, as well as the size and shape of adsorbed viruses. Figure 1A-C presents high resolution images of individual virus particles adsorbed onto several surfaces (pixel size ≈ 5 nm). These data are used for obtaining information on virus size, shape and deformation. To this end, it is necessary to understand the dilation effect due to the size of the AFM tip (Villarrubia 1997). In particular, the AFM tip passes through the virus structure in contact as the white cane of a blind person, obtaining dilated topographical details as small as the tip radius. In the case of a virus particle (Fig. 1D), the spherical shape of the virus is dilated by the parabola of the tip. Sometimes, luckily, the existence of a little tip prolongation at the tip apex would allow to resolve the individual protein capsids. By considering this effect, it is possible to evaluate the radius and contact angle of individual virus particles upon adsorption on each material class. Larger AFM images (8 μm × 8 μm) using lower resolution (pixel size ≈ 30 nm) as those in Fig. 1E-G, were used to quantify viral capture. The combination of both analyses reveals a surprising result. Specifically, the differences in virus capture between different materials are not correlated to the virus height or deformation upon capture on the surface. For example, the adsorption of virus particles on MoS_2_ leads to minimally deformed virus structures, although this surface captures ~ 15 times more viruses than other materials that show less deformed virus structures (García-Arribas et al. 2024). By using coarse grain simulations of the TGEV structure with an extended Helfrich theory for modeling the bending of the capsid, the experiments can be interpreted to suggest the complementary adhesion roles of the virus lipid shell and the protein spikes (Fig. 2AB). In brief, the mobility of spikes on the virus lipid shell allows the adhesion of CoV structures to be complementary driven either by the lipid shell or the spikes (Fig. 2BC).

## Biomechanics of tobacco mosaic virus (TMV)

**Fig. 3:**
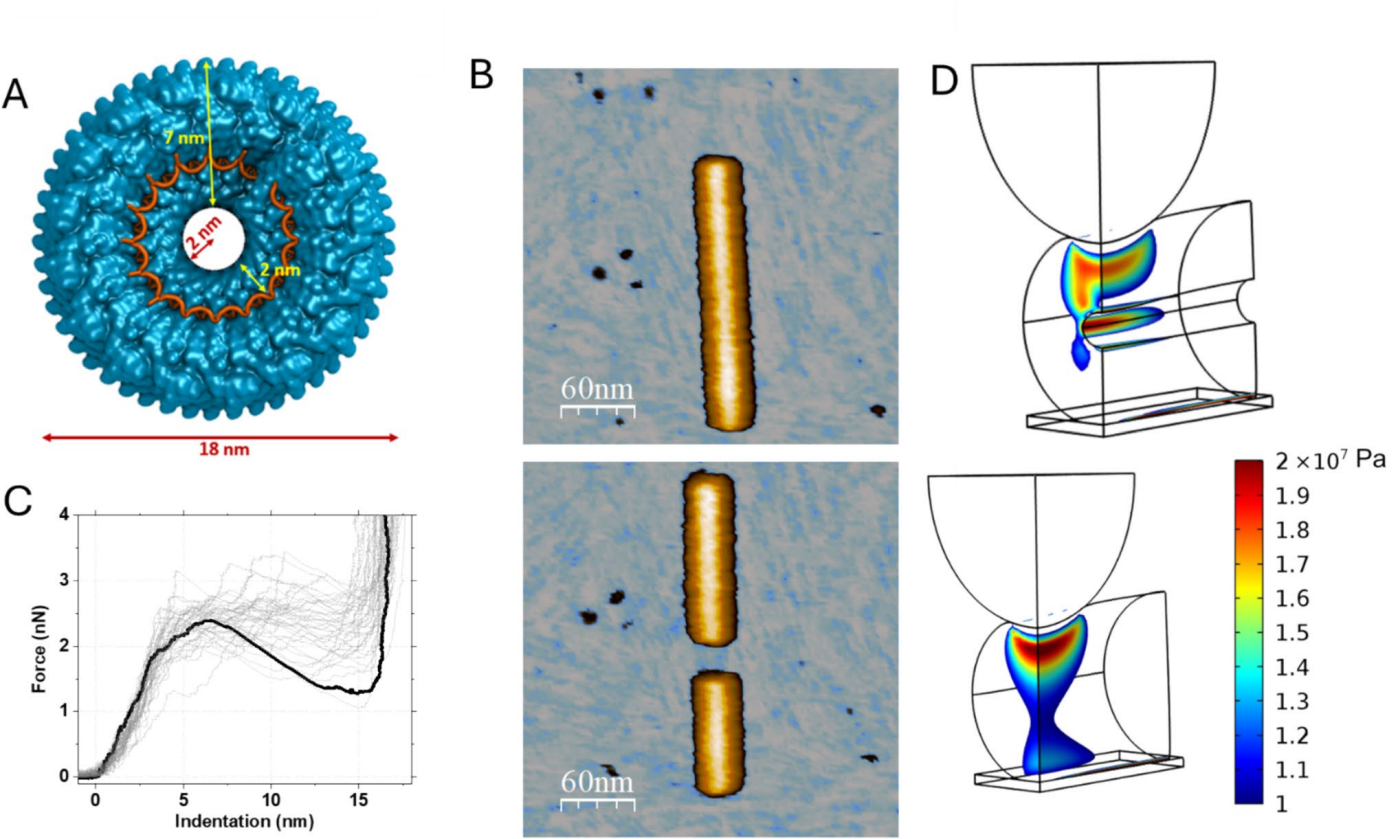
**A** Axial view of the TMV 3D model showing the helical protein capsid as well as the ssRNA inside, obtained from PDB 4UDV. **B** AFM image of a single TMV before (top) and after (bottom) single indentation assay. **C** 39 indentations performed on 39 viruses, one enhanced in dark color for the sake of clarity. **D** Finite element simulations. (Top) Nanoindentation in a hollow cylinder with an outer radius of 9 nm and an inner radius of 2 nm. (Bottom) Nanoindentation in a solid cylinder with an outer radius of 9 nm. In both cases, the indentation is 2.7 nm and the von Mises stress is indicated by colored contours and filtered to show only values greater than 1 × 10^7^ Pa. Adapted from (Díez-Martínez et al. 2025)

TMV has long been a preferred research model due to its simple helical architecture, remarkable stability, and ease of purification (Eleta-Lopez and Calò 2017; Creager 2022; Wege and Koch 2020). It has recently gained interest in bioengineering (Creager 2022) for uses in energy storage (Bittner et al. 2013), as a scaffold at the nanoscale (Young et al. 2008), and in drug delivery (Scholthof 2004). The TMV structure consists of a tubular protein coat approximately 300 nm in length, with an outer diameter of 18 nm and a central cylindrical cavity measuring 2 nm in radius (Fromm et al. 2015). The tubular shell is built of 2130 copies of the TMV Coat Protein (CP) organized in a helical structure. A single-stranded RNA molecule is lining the inner protomers of the protein capsid, which is located 4 nm from the center of the tubular structure (Fig. 3A).

### Single indentation assay

Single indentation assay is the initial approach for mechanical characterization. After selecting a TMV particle (Fig. 3B, top), the tip is positioned at the upper apex of the tube and moved at 50 nm/s, allowing water to escape from the virus as it is compressed (Zink and Grubmüller 2009). After the tip makes the initial contact with the TMV surface, the indentation plot shows an almost linear response (Fig. 3C, between 0 and 4 nm), which is ascribed to the elastic regime of the TMV structure. When critical indentation is reached, the particle breaks (Fig. 3C, 2.5 nN), showing a drastic fall of the force that ascribes to the tip apex passing through the virus structure (Fig. 3C, from 5 to 15 nm; Fig. 3B, bottom). Subsequently, the indentation data shows an infinity slope due to the presence of the solid supporting surface. Fitting the slope of the linear regime, the average stiffness of the TMV tubes is estimated to be 0.7 ± 0.2 N/m, with a breaking force of 2.5 nN. The value of the spring constant helps with adjusting a Finite Element Model. COMSOL Multiphysics Finite Element software was used for simulating the TMV indentation with two different structures: (i) a hollow tube (with internal and external radius of 2 nm and 9 nm, respectively, representing the TMV tube (Fig. 3D, top), and (ii) a solid cylinder with the same external radius (Fig. 3D, bottom). Both structures, with a length of 80 nm, were placed on a stiff surface and pushed by a rigid sphere (radius 15 nm) representing the AFM tip. Using symmetrical planes, the model was simplified to a quarter and meshed with ~ 50.000 quadrilateral elements. Deformation was calculated by solving nonlinear elasticity for several established tip positions, assuming Poisson’s ratio of 0.3. The experimental data were fitted by using the hollow cylinder response with Young’s modulus of 0.14 GPa (Fig. 3C). Finite Element modeling indicates that the mechanical stress is blocked by the cavity from reaching the bottom half of the tube in the case of the hollow cylinder (Fig. 3D, top). Conversely, the mechanical stress goes all the way down to the bottom of the tube in the case of the solid tube (Fig. 3D, bottom). This result appears to be interesting from a materials science point of view. However, it is pertinent to explore whether it might have implications during TMV disassembly, as we explain in the following section. In another context, single indentation assay has been also used for probing the flexibility changes induced by glycan in papillomavirus (Feng et al. 2024).

### Mechanical fatigue on individual virus particles

**Fig. 4:**
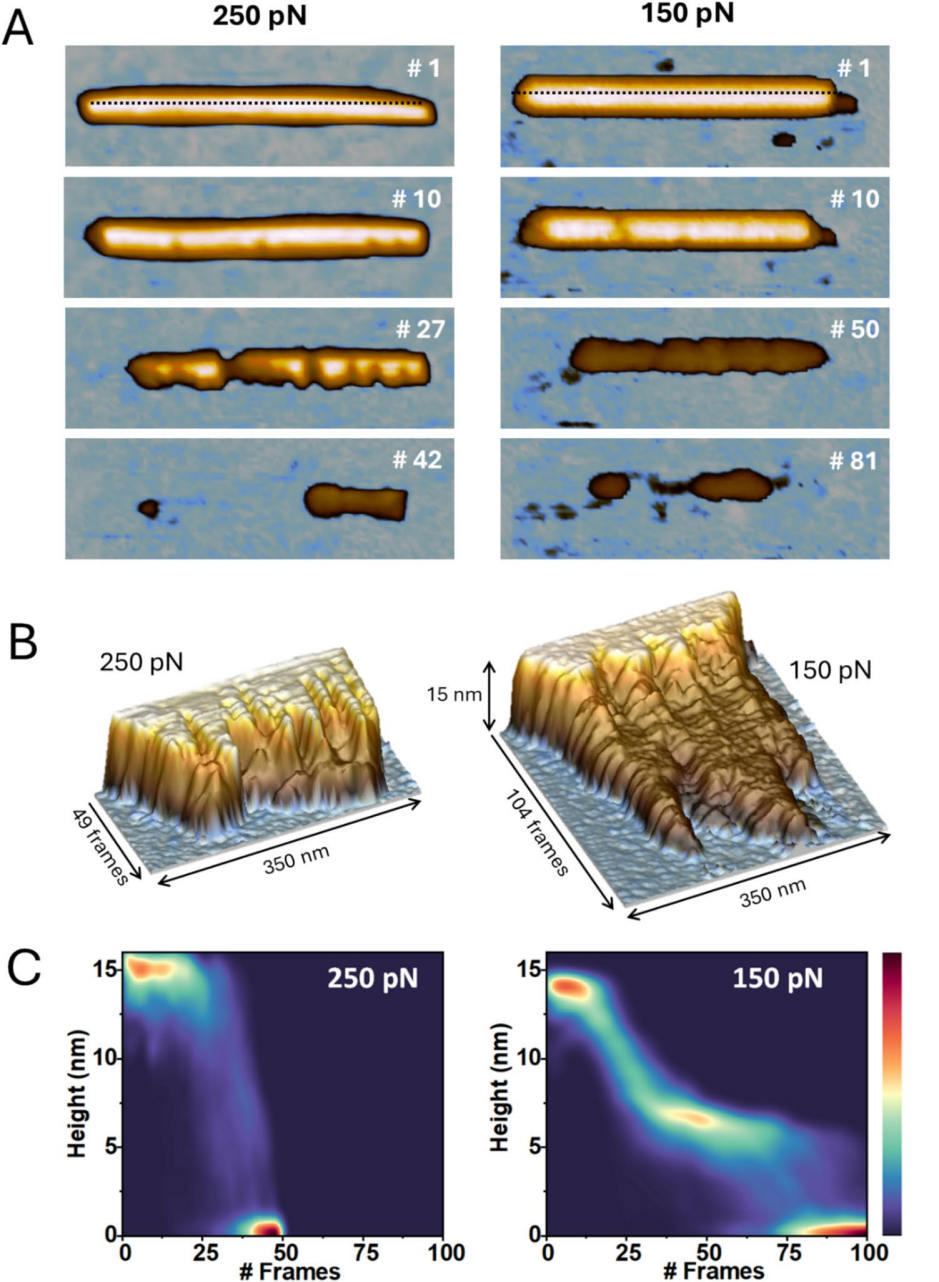
TMV disassembly with mechanical fatigue. **A** Sequence of AFM images at 250 pN (left) and 150 pN (right), indicating the frame number from top to bottom. **B** Kymograms showing the evolution of the central longitudinal profiles marked with dashed lines at frames #1. D. Density plots of the kymograms over disassembly to show the resistance of the virus at certain heights over time. Red areas indicate a higher concentration of points while indigo areas indicate the opposite. Adapted from (Díez-Martínez et al. 2025)

When using jumping plus mode (Ortega-Esteban et al. 2012), the imaging is achieved by moving the tip towards the sample until a specific force is measured. Subsequently, the z-piezo moves away the tip and shifts to the neighbor pixel to repeat the procedure. In this way, the AFM accomplishes a topographical drawing of the TMV particle by using a force well below the breaking force (~ 2.5 Nn, Fig. 3C). Virus structures can drop single protein capsomers through recurrent imaging at hundreds of pN, generating disassembly and genome release (Martin-Gonzalez et al. 2021; Rodríguez-Espinosa et al. 2023). Using mechanical fatigue in TMV generates rifts along the capsid structure (Fig. 4A, #10) that develop over time until the capsid is gone (Fig. 4A, bottom). The distance between rifts is too big to be related to individual coat proteins (CPs), and the size of the remaining virus pieces does not appear to be regular enough to show a quasi unit structure. However, the position of rifts along the virus could be related to the strength of the RNA-CP interaction. Although cracks happen at both high (250 pN) and low forces (150 pN), the disassembly dynamics differs for each case. AFM images are used to calculate kymograms that demonstrate the alteration of virus topography over the successive images. The AFM tip progressively wears down the virus particles, initially reflecting the loss of individual or small groups of coat proteins. Continued scanning enlarges these rifts by releasing adjacent CP subunits, resulting in an unzipping-like process. Under high forces (≥ 250 pN), CP loss occurs abruptly once rifts merge (~ 40 frames, Fig. 4B, left), and the virion disappears after ~ 50 frames. In contrast, at lower forces (~ 150 pN), disassembly proceeds gradually, with complete CP removal after ~ 100 frames (Fig. 6B, right). To emphasize these contrasting dynamics, kymogram data were converted into density plots (Fig. 4C), excluding frames prior to rift formation to focus on disassembly. These plots are obtained from the topography profile traced at the very top of the tube. For instance, in the first frames, the red color indicates that there are many pixels at ~ 15 nm of height because the virus structure remains almost intact at the beginning. The plots reveal a clear difference: at high forces, disassembly occurs in a single step from intact to collapsed structures, whereas at low forces, an intermediate state emerges at mid-height of the virion (Díez-Martínez et al. 2025). These observations suggest that the cylindrical cavity partially shields the lower half of the tube from applied force, consistent with predictions from the finite element model.

### Mechanical fatigue on two dimensional (2D) viral protein lattices

**Fig. 5:**
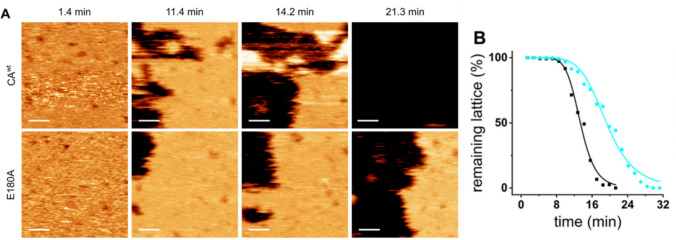
**A** Wild type (top) and E180A mutant (bottom) immunodeficiency CPs’ arrays on mica during mechanical fatigue experiments. The sequence of images shows how the CPs are gradually removed from the surface by mechanical fatigue. **B** Charts of the CPs’ coverage for wild type (black) and E180 mutant (cyan). Adapted from (Escrig et al. 2025)

Mechanical fatigue has also been applied for probing 2D arrangements of viral proteins. In particular, there are viruses whose subunits are prone to be arranged in 2D arrays. In the case of immunodeficiency virus, CPs can self-assemble into a 2D nanoarray that can coat large surfaces (Escrig et al. 2025). In that work, a genetic engineering strategy was used to remove electrostatic repulsion between the CPs forming the nanoarrays, and AFM was employed to probe the differences in mechanical stability (Escrig et al. 2025). Beyond finding differences between the spring constants found with single indentation assay, mechanical fatigue also produced interesting results (Fig. 5). An area of 400 nm^2^ was scanned cyclically under a force of ~ 40 pN for 2D arrays of wild type (Fig. 5A, top) and E180A mutant (Fig. 5A, bottom) with reduced electrostatic repulsion. From these mechanical fatigue experiments, the coverage of the viral CPs was monitored (Fig. 5B) for wild type (black) and E180A (cyan). These data show evidence that the mutant offers a higher resistance to fatigue as a directo consequence to the removal of the electrostatic repulsion between CPs.

## Genome release from individual minute virus of mice (MVM) virus particles

**Fig. 6:**
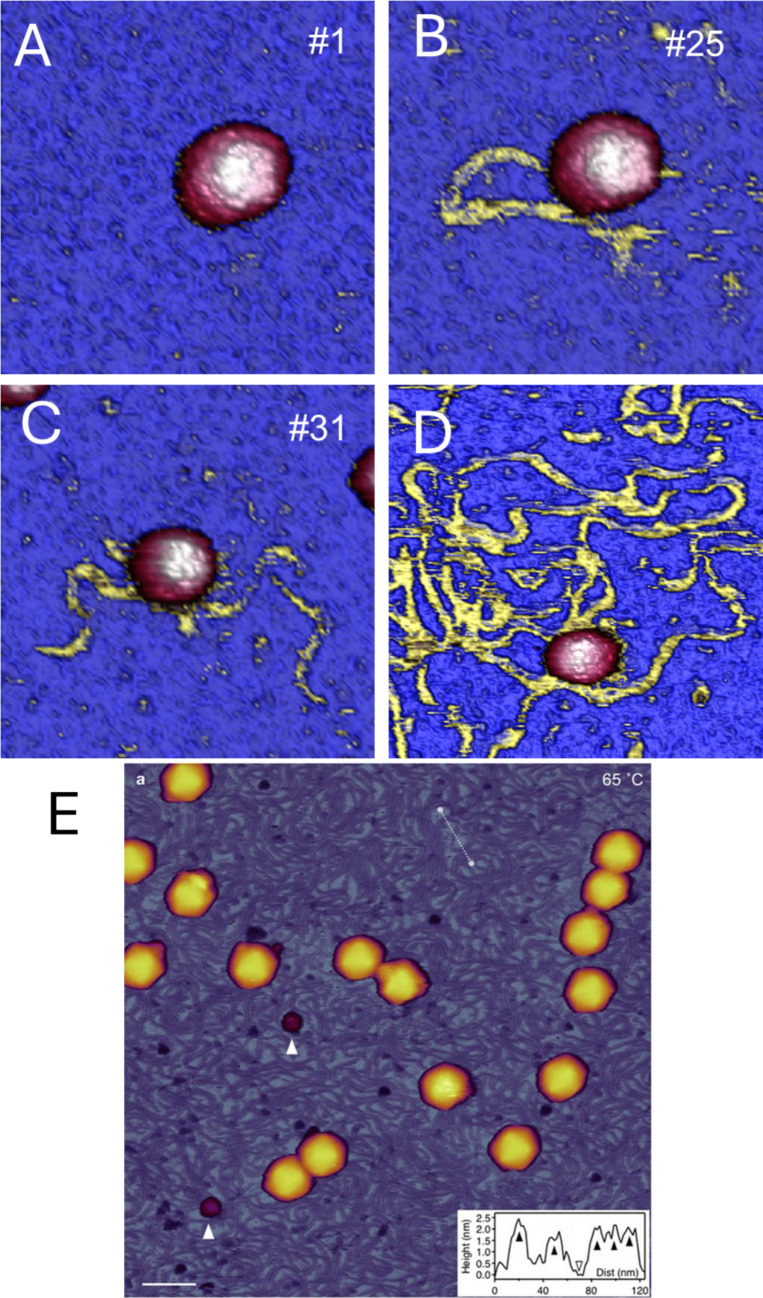
**A**, **B**, and **C** AFM topographies of MVM virus particles after 1, 25, and 31 consecutive frames, respectively. Image size is 200 nm × 200 nm for **A** and **B**, and 300 nm × 300 nm for **C**. **B** and **C** show the externalization of the viral genome in the form of a filament (yellow). **D** AFM image of a virion heated at 50 °C during 5 min, showing the capsid (pink) and the ejected genome (yellow). Adapted from (Strobl et al. 2023). **E** AFM of T7 phages treated at 65 ^°^C. Scale bar, 100 nm. White arrowheads point at large (10 nm) globular particles, probably capsomers. (Inset) Topographical height profile along the white dashed line. Black arrowheads point at DNA cross sections, whereas the empty arrowhead points at the mica support. Adapted from (Vörös et al. 2018)

MVM is among the smallest and simplest icosahedral viruses, measuring approximately 25 nm in diameter (Cotmore and Tattersall 2014). Its virion comprises an icosahedral capsid built from 60 protein subunits arranged into 20 trimeric building blocks (Reguera et al. 2005; Riolobos et al. 2006). The capsid features spicules or protrusions at the three-fold symmetry (S3) axes, cylindrical channels at the five-fold (S5) axes (through which the single-stranded DNA (ssDNA) genome is released), and annular depressions at the two-fold (S2) axes between cylinders and spicules (Llamas-Saiz et al. 1997; Agbandje-McKenna et al. 1998). MVM encapsidates ~ 5 kb of ssDNA without any apparent structural order(Agbandje-McKenna et al. 1998), although some segments are affixed to wedges of the inner wall, and close to the two-fold symmetry axes (Kontou et al. 2005) with important mechanical consequences (Carrasco et al. 2006). MVM virus particles were immobilized on a mica surface that was previously functionalized with poly-l-lysine (Strobl et al. 2023) and genome externalization was induced with both mechanical and thermal stress. In the first case, mechanical fatigue was applied in intact MVM virions (Fig. 6A) by AFM jumping mode plus, as explained before. Long and thin filaments with dimensions compatible with the diameter of an ssDNA molecule were detected around the MVM virus capsid (Fig. 6B and C). The virus particle did not present any alteration in height or morphology after the ejection of ssDNA. This suggests the absence of fractures of lost protein subunits and that the genome was ejected through the pores. Genome externalization was also induced by using thermal stress. In particular, the temperature increased to 50 °C and virus particles ejected a large amount of genome (Fig. 6D). The ejected ssDNA appears to have a larger persistence length than what ssDNA is supposed to have. In fact, the topo profile across the filaments (Strobl et al. 2023) indicates height values between 2 and 3 nm. This diameter is too thick for a single ssDNA fiber, even for dsDNA when observed with AFM (Martin-Gonzalez et al. 2019). Genome ejected with thermal cues (Figure 6D) shows a rich branching structure of ssRNA (yellow), whose fibers join and diverge on the surface. As it happens with ssRNA, we suggest the branching between ssDNA segments through complementary base pairs results in a stiffer genome structure that would have a larger persistence length. Thermal ejection has been also observed in dsDNA virus. In the case of T7 bacteriophage (Kellermayer et al. 2018; Vörös et al. 2018), the increase of temperature up to 80 ^0^C induced DNA ejection from virus particles (Fig. 6E). Thus, both mechanical fatigue and heating supply sufficient energy to weaken the ssDNA–capsid interactions and destabilize its tertiary and secondary structures, enabling ssDNA to diffuse through a single capsid pore or missing capsomer. Thermal stress has been also used to explore the resistance of TMV virus particles to high temperatures (Aldave et al. 2025). It was found that TMV can withstand a temperature up to 175 °C with minimum degradation, supporting once more the promising applications of this virus in materials science.

## Nanotribology of viruses

**Fig. 7:**
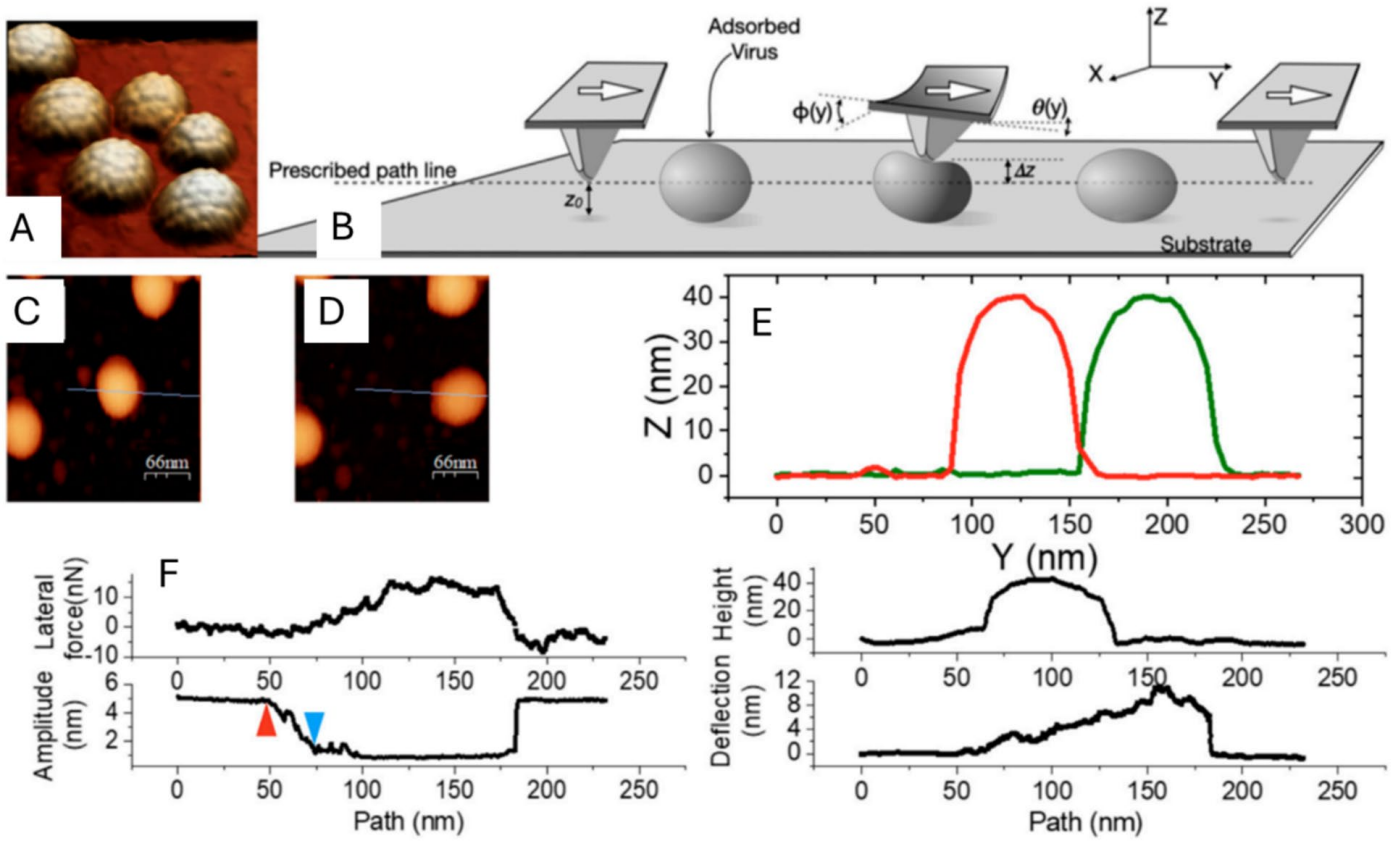
Principles of lateral force manipulation, illustrated with typical data for MPyV particles, adsorbed on HOPG, in buffer. **A** AFM topography of a group of MPyV capsids. **B** Cartoon of the lateral force manipulation experiment. ϕ is the torsion angle, while θ is the deflection angle. Δz is the linear deflection associated with θ. z0 is the tip position above the surface, on the prescribed path parallel to the substrate. **C** and **D** Images before and after manipulation of a virus particle by the tip moving along the blue line. (The slightly elongated aspect of viruses in **C** is a thermal drift artifact.) **E** Topographic plot profiles of the particle before (green) and after (red) manipulation. **F** Lateral force, oscillation amplitude, topography, and deflection profiles for a particle undergoing displacement. The red marker is where contact between the tip and virus occurs, and amplitude starts dropping. The blue marker points to the moment where the amplitude is suppressed–possible where the break between static and dynamic friction occurs. Adapted from (Ault et al. 2025)

**Fig. 8:**
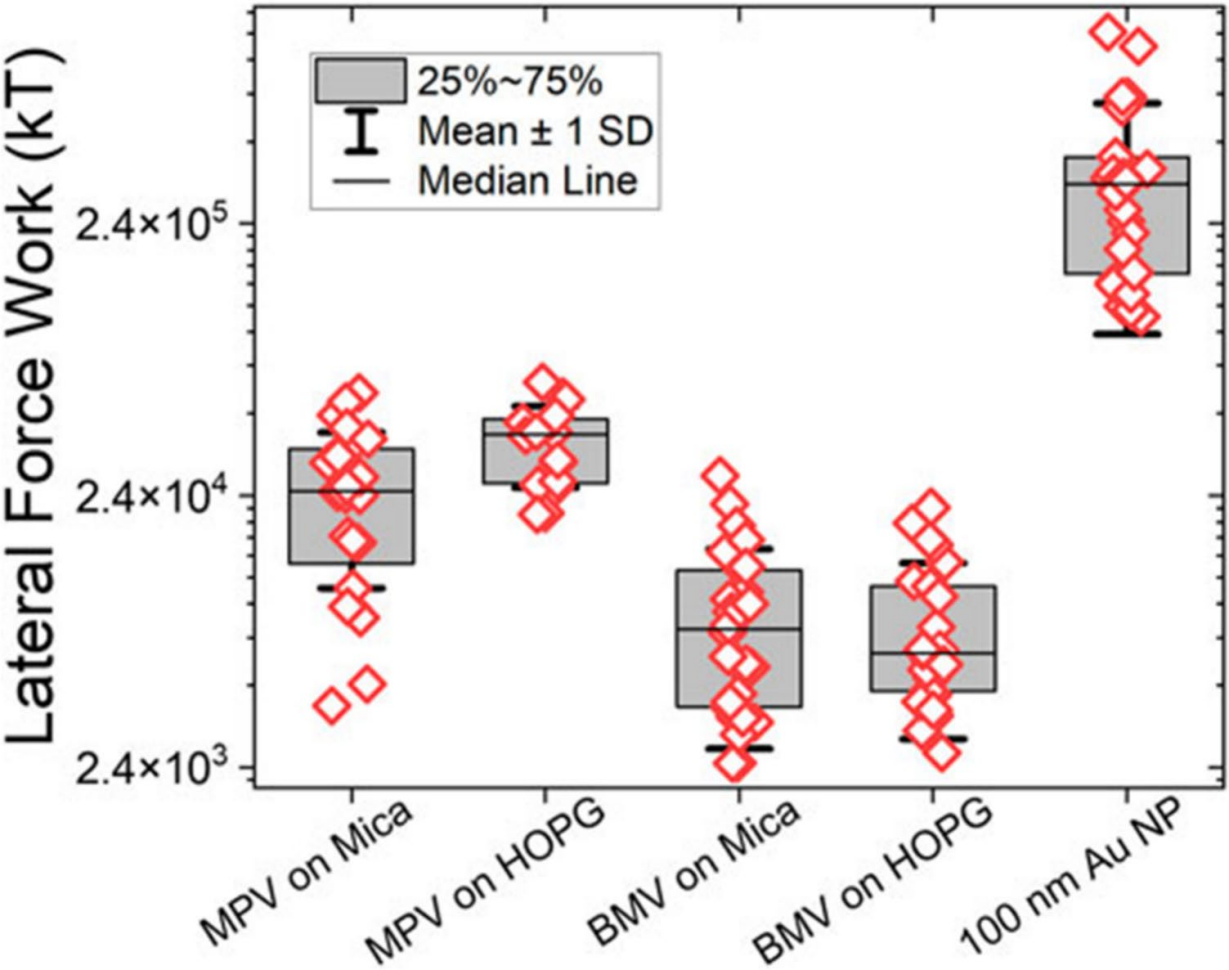
Experimental results for the work exerted by lateral force during manipulations. Adapted from (Ault et al. 2025)

When adsorbed on a surface, the non-rigid virus particles are partially deformed by the adhesive interaction with the surface, increasing the area of contact in comparison to rigid particles (Zeng et al. 2017; Rimai et al. 1994). With this respect there are simple but important issues that remain unexplored. When landing on a cell’s wall, how much of the virus transport on the surface is occurring by sliding vs. rolling until they find an access spot? Although at microscopic scale, the adhesion of solid nanoparticles at the air/surface interface has been investigated (Eppler et al. 2000; Cooper et al. 2001; Oras et al. 2018), the adherence of soft particles in liquid conditions remains poorly explored. The lateral manipulation abilities of AFM can address this gap (Falvo et al. 1997; Moya et al. 2020). For surface-adsorbed particles where constitutive interactions are comparable in magnitude to adhesion forces, the particle’s equilibrium shape arises from a balance between adhesive and elastic interactions (Piersanti et al. 2022). Contact mechanics is devoted to explore this energetic balance between these competing interactions (Johnson 1985; Israelachvili 2011). The tiny dimension changes of an adhered virus particle with respect to its nominal shape can be thoroughly studied in situ by the topographic images obtained with AFM (Zeng et al. 2017). This work focuses on studying three distinct nanoparticles. Two different compliant virus particles: brome mosaic virus (BMV) and murine polyoma virus (MPyV) with diameters of 28 nm and 45 nm, respectively. For the sake of comparison, stiff gold nanoparticles of various sizes are also manipulated. Figure 7A shows a few MPyV particles in liquid milieu dispersed on mica, where resolution is good enough to discern individual capsomers. The lateral manipulation procedure takes place as follows. First, a topographic image of the particles captured on the surface is obtained. A particle showing convenient dimensions is chosen to be manipulated. The “MicroAngelo” tool of the AFM software is used to carry out a typical AFM tip scan line of about 200 nm in length in the perpendicular direction to the long axis of the cantilever (dashed line, Fig. 7B), passing through the center of the selected capsid (blue line in Figs. 7C,D). The tip pre-scans the virus through this route in tapping mode, and a topographical profile is recorded. For the manipulation the tip path is repeated through the capsid in dynamic mode, but with a tip height over the surface (z_0_ ~ 4 nm) below the particle diameter for guaranteeing the contact between the tip and the particle, turning the feedback off. As the tip followed this path, the normal and torsional deflections, and the amplitude were recorded as a function of the probe position (Fig. 7F). Right after manipulation, a new image was recorded for quantifying the alterations in capsid location and/or structure. The comparison between the pre-manipulation and post-manipulation topographies (Figs. 7C, D) allows to monitor alterations in both particle position and shape (Fig. 7E). During the lateral manipulation experiment, the tip is set with an oscillation amplitude of ~ 5 nm at resonance frequency. The variation of the amplitude of this oscillation apprises on the fashion of the interaction between the particle and the AFM-tip (Fig. 7F, bottom left). The oscillation amplitude reduces to zero when the tip establishes a stable contact with the particle. Conversely, this oscillation reduction is only partial if the tip-particle contact is not stable. In this way, the oscillation of the cantilever informs of the tip-particle contact quality, thus indicating possible particle deviations from the prearranged manipulation line, as it happens when the particle spins away from the tip). It is possible to quantify the lateral work by integrating the lateral force (Fig. 8), resulting in∼ 10 times larger work values for Au NPs than for viruses. This result is in agreement with the differences of the Hamaker constant for proteins ($2.0\times {10}^{-20}$ J) (Roth et al. 1996) and Au NPs ($1.8\times {10}^{-19}$ J)(Pinchuk and Jiang 2015). It is possible to estimate the coefficients of areas of contact and interfacial energies by using the Johnson, Kendall, and Roberts (JKR) theory of contact mechanics (Johnson 1985). For Au NPs and MPyV and AuNPs and BMV on HOPG, the coefficient between the interfacial energies is 1.9 and 2.0, respectively (Ault et al. 2025). Therefore, by using the dimensions and mechanical parameters of each particle, the interfacial energy of Au NPs is estimated to be twice larger than that of virus particles. Remarkably, the area of contact of BMV and MPyV with the surface is 4 and 9 times larger than for Au NPs, respectively. Thus, virus particles remain less adhered than large and rigid Au NPs, even though soft viruses deform on the surface increasing their area of contact significantly with respect to the Au NPs. Consequently, the higher interfacial energy of Au NPs compensates for the geometrical features of area of contact and size.

## Pulling experiments: monitoring virus transmission and hydrophobicity

Viruses must traverse the host wall to deliver their genome. After genome replication and expression, newly assembled virus particles must again cross the cell membrane to exit the host for going on with the infection process. Many viruses exploit the endocytic pathway for entry (Bamford and Zuckerman 2021). Before internalization, they must first attach to the plasma membrane through specific interactions with cellular receptors (Yang et al. 2021). A well-known example is the binding between HAdV fibers and CAR proteins at the plasma membrane (José Gallardo et al. 2021). Atomic force microscopy (AFM) enables direct measurement of the forces involved in these interactions (Ray et al. 2024). In such experiments, individual virus particles are covalently linked to the AFM tip. When combined with confocal microscopy, this setup allows precise co-localization of cultured cells with the AFM tip. Consequently, the tip can scan targeted regions of the cell surface to quantify the adhesive forces between the virus particle and membrane receptors (Alsteens et al. 2017). Recording these interactions provides valuable insight into the mechanics of viral attachment. Monitoring adhesion over time revealed discrete binding events between the virus particle and its receptor. These force spectroscopy experiments enabled the determination of key parameters such as the dissociation rate constant and the free energy of the fiber–receptor interaction. Pulling experiments have also been used to measure the hydrophobicity and charge of individual virus particles. In the case of Adeno-associated virus (AAV), the AFM tip was functionalized with either hydrophobic or charged molecules, and the adhesion force between the tip and the virus was explored (Heldt et al. 2023). This experiment was able to differentiate between empty and full AAV particles because they presented different adhesion forces. 

## Conclusion and perspectives

The first viral structure explored with a scanning probe technique was bacteriophage phi29 (Baró et al. 1985), predating the creation of atomic force microscopy (AFM) by one year (Binnig et al. 1986). During the subsequent years, AFM became a widely adopted method for investigating biological structures at the single-molecule level (Bustamante et al. 1992). AFM was initially used for imaging virus particles (Kuznetsov et al. 2001; Drygin et al. 1998), but its applications soon expanded to include investigations of virus biophysics (de Pablo 2024). Over the past two decades, AFM has gradually gained recognition as a valuable tool in virology, although it has not yet reached the prominence of classical structural techniques such as electron or X-ray microscopy. In this vein, AFM is ascribed as providing complementary information to the classical techniques. Although AFM cannot compete in structural resolution with X-ray and electron microscopy, it is unique for working in liquid conditions and in real time with the same virus particle. AFM can apply and measure forces in liquid milieu, allowing the buffer to change at will (Cantero et al. 2022). The manipulation ability provided by the nanometric tip results in a powerful nanosurgery technique that allows the exploration of both the capsid and viral nucleic acids (Martin-Gonzalez et al. 2021, 2019; Kellermayer et al. 2018; Cantero et al. 2023; Hernando-Pérez et al. 2019). A promising development to monitor the real-time assembly disassembly dynamics of two-dimensional virus-like structures (Fig. 5) is High Speed AFM (HSAFM) (Valbuena et al. 2020). However, a big challenge for HSAFM is to monitor the assembly of 3D virus structures on the surface. The combination of AFM with other single molecule techniques, such as total internal reflection microscopy (TIRFM), allows the simultaneous exploration of virus particles with fluorescence and AFM (Strobl et al. 2022; Ortega-Esteban et al. 2015; Valbuena et al. 2024). All these advances may help establish AFM as a technical approach for virus research that is valid and accepted between specialists as the classical structural techniques. We hope that the applications presented in this revision will help to reach this goal.

## Data Availability

No datasets were generated or analyzed during the current study.
